# Supplementary material for: On the Degree of Plastic Strain during Laser Shock Peening of Ti-6Al-4V
Source: Materials (Basel). 2023 Jul 30;16(15):5365. doi: 10.3390/ma16155365 (PMC10419942; doi:10.3390/ma16155365)
Supplement: Supplementary file 1 [file materials-16-05365-s001.zip › materials-2508720-supplementary.docx]

**Evaluation of thermal effects induced during laser shock peening**

***1. Broad aspects of laser shock peening***

Laser shock peening (LSP) is an innovative technique that is applied to enhance the fatigue strength of structural materials. It involves high-energy laser pulsing of the metal surface, which typically results in the generation of residual compressive stresses.

The schematic diagram of the LSP working principle is shown in Figure 1. Prior to LSP, the processed surface is usually covered with an opaque sacrificial overlay (for example, aluminum or steel foil). Moreover, running water is also typically used as a transparent confining medium. The propagation of a laser pulse across the interface between two media with distinctly different densities (i.e., the water layer and the sacrificial overlay) normally results in a plasma spot. The rapid expansion of the plasma gives rise to shock waves, which, in turn, exert a pressure effect [1, 2]. At a pressure level greater than the Hugoniot elastic limit, the processed material experiences a plastic strain. Due to the intrinsically inhomogeneous character of the distribution of such strain, it results in residual stress.


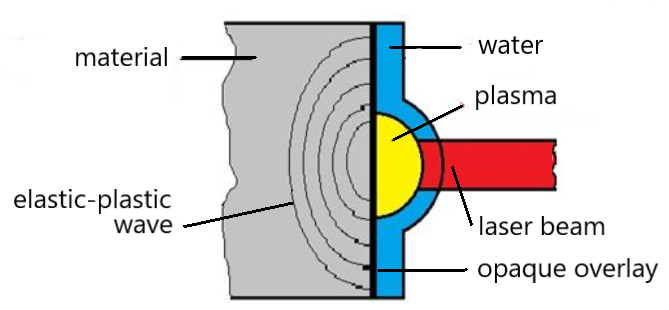


**Figure S1**. Schematic illustration of LSP (after Amarchinta [2])

An important issue of the LSP is its extremely short processing time. Specifically, the typical duration of the laser pulsing is only ~10 ns, while the estimated lifetime of the plasma is ~ 100 ns. Hence, it is believed that the activation of diffusion-driven processes is unlikely. Thus, it is widely accepted that the processed material remains relatively cold during LSP [3-40]. Wu [41] was one of the first to suggest that LSP-induced stresses are mechanical rather than thermal in nature. Therefore, LSP is often considered a mechanical rather than a thermal process [1].

***2. Plasma spot. Characteristic size***

It is sometimes believed that the sacrificial layer during LSP serves two functions: (i) prevention of the processed material from the ablation effect and (ii) protection from the direct influence of the plasm spot. The latter issue is considered in the present section. To this end, a characteristic thickness of vaporized material was estimated.

During high-energy laser pulsing, the total energy absorbed by a processed material may be evaluated as , where is heating energy, is melting energy, and is vaporization energy.

Neglecting material heating in the liquid and gas phases and assuming that the total absorbed energy is converted into heat, this equation can be rewritten as , where is heat capacity (taken to be 460 J/(kg×К)), is material density (taken to be 7800 kg/m3), is the volume of vaporized material (~, here is the minimum characteristic size of a laser spot), is a temperature difference between ambient condition and the melting point (taken to be 1800 K), is the specific heat of fusion (taken to be 2000 J/сm3 or 2.7×105 J/kg), and is the specific heat of vaporization (taken to be 43000 J/сm3 or 6.3×106 J/kg).

Hence, the characteristic thickness of the vaporized material is .

Given a total absorbed energy of 10 J, the upper limit of the characteristic thickness of the vaporized material should be ~ 10-4 m. This value is comparable with the thickness of the coating layer. Therefore, the simple estimations given above are in good correspondence with the initial presumption that the plasma spot is confined within the sacrificial material.

***3. Plasma spot. Characteristic time and induced pressure***

The evaporation process can be considered in terms of the propagation of an evaporation wave. The wave velocity should increase with the temperature of the vaporized material, but then it is expected to saturate at the magnitude of , where is the radiation power density. After a characteristic time (where is thermal diffusivity; taken to be 1×10-5 m2/s), the evaporation wave outraces the heat conduction wave, thus diminishing the role of the latter process in material heating. Hence, to provide material evaporation, the applied laser power density should exceed ~108 W/cm2. At the energy density of 109 W/cm2, which is characteristic of the LSP treatment, the evaporation process occurs without a phase transition, and the radiation energy is almost completely absorbed by the ionized gas.

The amplitude of the pressure associated the plasma expansion can be estimated on the basis of a one-dimensional plasma model [42]. According to this model, this pressure remains even after laser pulsing, and the duration of the “stressed” condition can significantly (~10 times) exceed the duration of the pulsing time.

It is important to emphasize that the interaction of the laser beam with the water layer (Fig. 1) may also give rise to plasma. It is opaque to the laser beam, and thus it may absorb a portion of the laser energy that exceeds a certain threshold. This situation has been considered in a number of works, which show that the threshold energy lies in the range of 8-10 GW/cm2 [e.g., 43]. Accordingly, the magnitude of the pulse-induced pressure was predicted to saturate at ~4 GPa and show only subtle changes with a further increase in laser power density [43]. The imposing of such pressure for a characteristic time of 100 ns should result in the generation of elastic-plastic waves rather than shock waves.

***4. Thermal effect of LSP***

An oversimplified evaluation of the heat propagation from the laser-shock-peened surface to the bulk material can be obtained from a consideration of its characteristic rate, where is time. Hence, the propagation depth of the heat transfer can be estimated as . Taking the characteristic duration of the LSP pulse (20 ns in the present case), this gives a depth of ~10-7 m. Considering the characteristic time of the LSP-“stressed” state (i.e., ~100 ns), the propagation depth should be of ~10-6 m.

A more thorough insight into this issue can be obtained by solving the one-dimensional heat conduction problem for a dimensionless temperature change of . In this approach, the normalized spatial scale is , and the normalized time scale is . The boundary-value problem for a homogeneous heat equation is specified as follows: Find the function , defined in the field , which satisfies the equation:

for

with an initial condition:

for .

and boundary conditions:

*,* for .

The solution of this problem is as follows

Consider the process of heating a material in contact with a plasma spot under the assumption that all the energy of laser radiation is absorbed by the ionized gas. At the contact time of 100 ns, the gas temperature at the energy density of ~109 W/cm2 should reach a magnitude of ~10 electron volts. Taking the temperature of the ionized gas to be ~2×104 K, the predicted evolution of temperature as a function of the propagation depth is shown in Figure 2. It is seen that the temperature rapidly drops below 0.5 Tm (where Tm is the melting point); i.e., to a level at which its influence on microstructural changes becomes minimal.

| 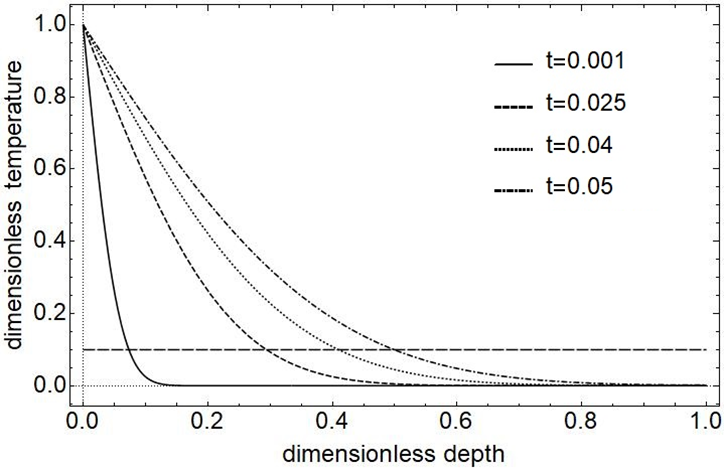 |
| --- |
| **Figure S2**. Evolution of temperature with the distance from the plasma spot as a function of time (t). Note: The horizontal line corresponds to 0.5 Tm, where Tm is the melting point. |

To obtain an additional insight into the temperature effect, the thickness of the material layer heated above 0.5 Tm was calculated as a function of time. The obtained results are shown in Figure 3. It can be seen that the thickness was only predicted to be a few microns.

| 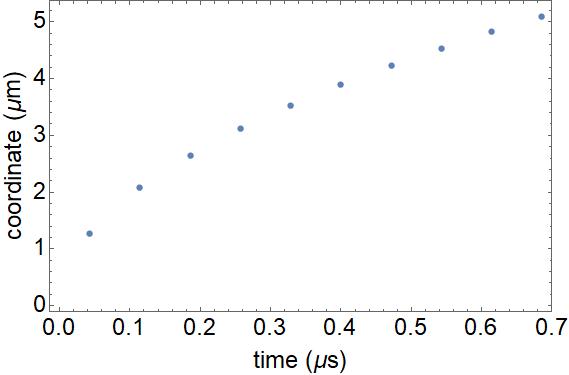 |
| --- |
| **Figure S3**. Dependence of the location of the point corresponding to half of the homologous temperature as a function of time when contacting an object with a temperature of 2 x 104 К. |

In this context, it is important to emphasize that the laser-shock-peened material is normally covered by a sacrificial (protective) layer. The typical thickness of this layer is ~100 microns, thus being ~100 times greater than the characteristic depth at which the thermal effect should be significant. Thus, it can be concluded that any thermal processes that are essential for microstructural modification of the pulsed material are limited by the thickness of the sacrificial (protective) layer.

**References**

[1] K. Ding., L. Ye, Laser shock peening: Performance and process simulation, Woodhead Publishing Limited, Cambridge, UK, 2006 162 p.; https://doi.org/10.1504/IJMMP.2008.018396

[2] H. Amarchinta, Uncertainty quantification of residual stresses induced by laser peening simulation. PhD Dissertation in Engineering, Dayton: Wright State University, 2010. 201 p.

[3] S. Ashley *Powerful laser means better peening*, Mech. Eng. 120 (1998) 12–12; https://doi.org/10.1016/S0142-1123(02)00022-1

[4] L. Berthe, R. Fabbro, P. Peyre, E. Bartnicki, *Wavelength dependent of laser shock-wave generation in the water-confinement regime*, J. Appl. Phys. 85 (1999) 7552–7555; https://doi.org/10.1063/1.370553

[5] L. Berthe, R. Fabbro, P. Peyre, L. Tollier, E. Bartnicki, *Shock waves from a water confined laser-generated plasma,* J. Appl. Phys. 82 (1997) 2826–2832; https://doi.org/10.1063/1.366113

[6] W. Braisted, R. Brockman, *Finite element simulation of laser shock peening,* Int. J. Fatigue 21 (1999) 719–724; https://doi.org/10.1016/S0142-1123(99)00035-3

[7] W. Zhang, YL. Yao, *Micro scale laser shock processing of metallic components*, J. Manuf. Sci. Eng. 124 (2002) 369–378; https://doi.org/10.1016/S1526-6125(01)70128-4

[8] H. Chen, Y. Wang, JW. Kysar, YL. Yao *Study of anisotropic character induced by microscale laser shock peening on a single crystal aluminum,* J. Appl. Phys. 101 (2007) 024904; https://doi.org/10.1063/1.2424500

[9] G.J. Cheng, M.A. Shehadeh *Multiscale dislocation dynamics analyses of laser shock peening in silicon single crystals*, Int. J. Plasticity 22 (2006) 2171–2194; https://doi.org/10.1016/j.ijplas.2006.03.006

[10] K. Ding, *Three-dimensional dynamic finite element analysis of multiple laser shock peening processes,* Surf. Eng. 19 (2003) 351–358; https://doi.org/10.1179/026708403225007563

[11] B.P. Fairand, A.H. Clauer, R.G. Jung, B.A. Wilcox *Quantitative assessment of laser-induced stress waves generated at confined surfaces*, Appl. Phys. Lett. 25 (1974) 431–433; https://doi.org/10.1063/1.1655536

[12] R. Fabbro, J. Fournier, P. Ballard, D. Devaux, J. Virmont, *Physical study of laser-produced plasma in confined geometry*, J. Appl. Phys. 68 (1990) 775–784; https://doi.org/10.1063/1.346783

[13] Y. Hu, Z. Yao, *Numerical simulation and experimentation of overlapping laser shock processing with symmetry cell*, Int. J. Machine Tools Manuf. 48 (2008) 152–162; https://doi.org/10.1007/s12206-012-1263-0

[14] C.S. Montross, T. Wei, L. Ye, G. Clark, Y.W. Mai *Laser shock processing and its effects on microstructure and properties of metal alloys: a review*, Int. J. Fatigue, 24 (2002) 1021–1036; https://doi.org/10.1016/S0142-1123(02)00022-1

[15] P. Peyre, L. Berthe, X. Scherpereel, R. Fabbro, *Laser-shock processing of aluminum coated 55C1 steel in water-confinement regime, characterization and application to high-cycle fatigue behavior*, J. Mater. Sci. 33 (1998)1421–1429; https://doi.org/10.1023/A:1004331205389

[16] P. Peyre, A. Sollier, I. Chaieb, L. Berthe, E. Bartnicki, C. Braham, R. Fabbro. *FEM simulation of residual stresses induced by laser peening,* Eur. Phys. J. Appl. Phys. 23 (2003) 83–88; https://doi.org/10.1051/epjap:2003037

[17] P. Peyre, R. Fabbro, P. Merrien, H.P. Lieurade, *Laser shock processing of aluminum alloys. Application to high cycle fatigue behavior*, Mater. Sci. Eng. A210 (1996) 102–113; https://doi.org/10.1016/0921-5093(95)10084-9

[18] R.A. Brockman, W.R. Braisted, S.E. Olson, R.D. Tenaglia, A.H. Clauer, K. Langer, M.J. Shepard, *Prediction and characterization of residual stresses from laser shock peening*, Int. J. Fatig., 36 (2012) 96–108; https://doi.org/10.1016/j.ijfatigue.2011.08.011

[19] P. Peyre, L. Berthe, R. Fabbro, A. Sollier, *Experimental determination by PVDF and EMV techniques of shock amplitudes induced by 0.6–3 ns laser pulses in a confined regime with water*, J. Phys. D: Appl. Phys., 33 (2000) 498–503; https://doi.org/10.1088/0022-3727/33/5/305

[20] P. Peyre, L. Berthe, X. Scherpereel, R. Fabbro, E. Bartnicki. *Experimental study of laser-driven shock waves in stainless steels*, J. Appl. Phys. 84 (1998) 5985–5992; https://doi.org/10.1063/1.368894

[21] P. Peyre, I. Chaieb, C. Braham, *FEM calculation of residual stresses induced by laser shock processing in stainless steels*, Modelling Simul. Mater. Sci. Eng. 15 (2007) 205–221; https://doi.org/10.1088/0965-0393/15/3/002

[22] A.W. Warren, Y.B. Guo, S.C. Chen, *Massive parallel laser shock peening: simulation, analysis, and validation*, Int. J. Fatigue 30 (2008) 188–197; https://doi.org/10.1016/j.ijfatigue.2007.01.033

[23] LSP Technologies, Inc., <http://lsptechnologies.com/>. Last accessed March 31 2012

[24] Metal Improvement Company, <http://www.metalimprovement.com/>. Last accessed March 31 2012

[25] Y. Fan, Y. Wang, S. Vukelic, Y.L. Yao, *Numerical investigation of opposing dual sided microscale laser shock peening*, J. Manuf. Sci. Eng. 129 (2007) 256–264; https://doi.org/10.1115/1.2540771

[26] H. Chen, J.W. Kysar, Y.L. Yao *Characterization of plastic deformation induced by microscale laser shock peening*, J. Appl. Mech. 71 (2004) 713–723; https://doi.org/10.1115/1.1782914

[27] W. Zhang, Y.L. Yao, I.C. Noyan, *Microscale laser shock peening of thin films, part 1: experiment, modeling and simulation*, J. Manuf. Sci. Eng. 126 (2004) 10–17; https://doi.org/10.1115/1.1645878

[28] K. Ding, L. Ye, Laser shock peening: performance and process simulation. 2006: Woodhead Publishing. https://doi.org/10.1201/9781439823620

[29] S. Gulshan, Effective Simulation and Optimization of a Laser Peening Process (2009). Wright State University, Theses and Dissertations. 954. https://corescholar.libraries.wright.edu/etd_all/954

[30] H. Amarchinta, R. Grandhi, K. Langer, D. Stargel, *Material model validation for laser shock peening process simulation*, Modell. Sim. Mater. Sci. Eng. 17 (2008) 015010; https://doi.org/10.1088/0965-0393/17/1/015010

[31] G. Singh, R.V. Grandhi and D.S. Stargel, *Modeling and parameter design of a laser shock peening process*. Int. J. Comp. Meth. Eng. Sci. Mech. 12(5) (2011) p. 233-253

[32] S. Keller, S. Chupakhin, P. Staron, E. Maawad, N. Kashaev, B. Klusemann, *Experimental and numerical investigation of residual stresses in laser shock peened AA2198*, J. Mater. Process. Technol., 255 (2018) 294-307; https://doi.org/10.1016/j.jmatprotec.2017.11.023

[33] T.J. Spradlin, R.V. Grandhi, K. Langer. *Experimental validation of simulated fatigue life estimates in laser-peened aluminum*, Int. J. Struct. Integr. 2 (2011) 74-86; https://doi.org/10.1108/17579861111108635

[34] K. Langer, T.J. Spradlin, M. E. Fitzpatrick, *Finite element analysis of laser peening of thin aluminum structures*, Metals, 10, (2020) 93-103. https://doi.org/ 10.3390/met10010093

[35] N. Hfaiedh, P. Peyre, H. Song, I. Popa, V. Ji, *Finite element analysis of laser shock peening of 2050-T8 aluminum alloy*, Int. J. Fatigue, 70 (2015) 480-489; https://doi.org/10.1016/j.ijfatigue.2014.05.015

[36] X. Zhang, H. Li, S. Duan, X. Yu, J. Feng, B. Wang, Z. Huang, *Modeling of residual stress field induced in Ti–6Al–4V alloy plate by two sided laser shock processing*, Surf. Coat. Technol., 280 (2015) 163-173; https://doi.org/10.1016/j.surfcoat.2015.09.004

[37] S. Boyu, Q. Hongchao, Z. Jibin, *Accurate numerical modeling of residual stress fields induced by laser shock peening,* AIP Advances, 8 (2018) 095203; https://doi.org/10.1063/1.5039674

[38] P. Mylavarapu, C. Bhat, M. K. Reddy Perla, K. Banerjee, K. Gopinath, T. Jayakumar, *Identification of critical material thickness for eliminating back reflected shockwaves in laser shock peening – A numerical study*, Opt Laser Technol., 142 (2021) 107217; https://doi.org/10.1016/j.optlastec.2021.107217

[39] C. Le Bras, A. Rondepierre, R. Seddik, M. Scius-Bertrand, Y. Rouchausse, L. Videau, B. Fayolle, M. Gervais, L. Morin, S. Valadon, R. Ecault, D. Furfari, L. Berthe, *Laser shock peening: Toward the use of pliable solid polymers for
confinement*, Metals 9 (2019) 793–806; https://doi.org/10.3390/met9070793

[40] M. Frija, M. Ayeb, R. Seddik, R. Fathallah, H. Sidhom *Optimization of peened-surface laser shock conditions by method of finite element and technique of design of experiments*, Int. J. Adv. Manuf. Technol. 97 (2018) 51–69; https://doi.org/10.1007/s00170-018-1849-5

[41] B. Wu, *Thermal Stress in Laser Shock Peening*. In: Hetnarski, R.B. (eds) Encyclopedia of Thermal Stresses. Springer, Dordrecht. https://doi.org/10.1007/978-94-007-2739-7_17

[42] X. Li, W. He, S. Luo, X. Nie, L. Tian, X. Feng, R. Li, *Simulation and experimental study on residual stress distribution in titanium alloy treated by laser shock peening with flat-top and Gaussian laser beams*, Materials, 12 (2019) 1343-1349; https://doi.org/10.3390/ma12081343

[43] B. Wu, Y.C. Shin, *Laser pulse transmission through the water breakdown plasma in laser shock peening* // Appl. Phys. Let. 88 (2006) 041116.
